# Potential Prodromal Digital Postural Sway Markers for Fragile X-Associated Tremor/Ataxia Syndrome (FXTAS) Detected via Dual-Tasking and Sensory Manipulation

**DOI:** 10.3390/s24082586

**Published:** 2024-04-18

**Authors:** Emily C. Timm, Nicollette L. Purcell, Bichun Ouyang, Elizabeth Berry-Kravis, Deborah A. Hall, Joan Ann O’Keefe

**Affiliations:** 1 Department of Anatomy & Cell Biology, Rush University Medical Center, Chicago, IL 60612, USA; emily_c_timm@rush.edu (E.C.T.); elizabeth_berry-kravis@rush.edu (E.B.-K.); 2 Department of Neurological Sciences, Rush University Medical Center, Chicago, IL 60612, USA; bichun_ouyang@rush.edu (B.O.); deborah_a_hall@rush.edu (D.A.H.); 3 Department of Pediatrics, Rush University Medical Center, Chicago, IL 60612, USA

**Keywords:** fragile X-associated tremor/ataxia syndrome (FXTAS), fragile X messenger ribonucleoprotein 1 (*FMR1*) gene premutation carriers, balance, dual-tasking, prodromal signs, instrumented SWAY

## Abstract

FXTAS is a neurodegenerative disorder occurring in some Fragile X Messenger Ribonucleoprotein 1 (*FMR1*) gene premutation carriers (PMCs) and is characterized by cerebellar ataxia, tremor, and cognitive deficits that negatively impact balance and gait and increase fall risk. Dual-tasking (DT) cognitive-motor paradigms and challenging balance conditions may have the capacity to reveal markers of FXTAS onset. Our objectives were to determine the impact of dual-tasking and sensory and stance manipulation on balance in FXTAS and potentially detect subtle postural sway deficits in *FMR1* PMCs who are asymptomatic for signs of FXTAS on clinical exam. Participants with FXTAS, PMCs without FXTAS, and controls underwent balance testing using an inertial sensor system. Stance, vision, surface stability, and cognitive demand were manipulated in 30 s trials. FXTAS participants had significantly greater total sway area, jerk, and RMS sway than controls under almost all balance conditions but were most impaired in those requiring vestibular control. PMCs without FXTAS had significantly greater RMS sway compared with controls in the feet apart, firm, single task conditions both with eyes open and closed (EC) and the feet together, firm, EC, DT condition. Postural sway deficits in the RMS postural sway variability domain in asymptomatic PMCs might represent prodromal signs of FXTAS. This information may be useful in providing sensitive biomarkers of FXTAS onset and as quantitative balance measures in future interventional trials and longitudinal natural history studies.

## 1. Introduction

Fragile X-associated tremor/ataxia syndrome (FXTAS) is a neurodegenerative disease characterized by cerebellar gait ataxia, action tremor, and executive dysfunction causing balance, gait, and mobility impairments, increased fall risk, and poor quality of life [1,2]. It is caused by a 55–200 CGG repeat expansion (termed a “premutation”) in the promotor region of the fragile X messenger ribonucleoprotein 1 (*FMR1*) gene located on the X chromosome [3]. Premutation alleles are associated with elevated levels of expanded CGG repeat-containing *FMR1* mRNA, which causes the activation of cellular stress pathways and RNA-mediated toxicity with CGG binding protein sequestration, repeat-associated non-AUG-initiated (RAN) translation [4], and subsequent neurodegeneration, especially in the cerebellum, cerebral cortex, and cerebellar–cortical pathways [5,6,7,8,9,10]. FXTAS is not fully penetrant and occurs in approximately 50% of men carriers and 16% of women carriers, generally beginning after age 50, with incidence increasing with age [11]. Women are less commonly and perhaps less severely affected because of the protective effects of the normal *FMR1* allele on their second X chromosome [3,12]. Advancing age [11] and increased CGG repeat size increase the risk of getting FXTAS [13], and CGG repeat length correlates with age of onset [14] and the severity of many phenotypic characteristics of the disease [13,15].

There are notable executive function deficits in FXTAS [16,17,18], which have been shown to make holding, shifting, and dividing attention between tasks extremely challenging in other neurodegenerative disorders [19]. As a result, dual-tasking (DT), where one performs a cognitive and motor task simultaneously, and other challenging balance and gait activities become increasingly difficult. The known prefrontal cortical (among other cortical regions) and cerebellar neurodegeneration and loss of white matter integrity in frontal-cerebellar circuitry [5,6,7,8,9,10] are likely to make DT during balance in FXTAS very challenging. We previously found significant DT cognitive–motor interference for turning in men with FXTAS, such that turn speed was reduced by the simultaneous performance of an executive function task [20]. However, no studies have investigated the effects of DT on balance performance in men and women PMC with and without FXTAS. In addition, more information on how DT and other environmentally challenging conditions impact balance in persons with FXTAS is necessary to inform neurorehabilitation strategies. The neural control of posture depends on the integration of sensory information received from the proprioceptive, visual, and vestibular systems and appropriate motor outputs to achieve adaptable postural control [21]. Postural conditions that are more challenging (for example, adding a dual cognitive task, standing on compliant surfaces that reduce accurate proprioceptive input, closing one’s eyes, and narrowing the base of support) require greater neural control mechanisms, which are typically impaired in persons with cerebellar disorders like FXTAS.

Examining postural control under conditions of altered sensory input and DT has the potential to translate findings into neurorehabilitation approaches designed to improve balance and gait in FXTAS. For example, tactile stimulation to the plantar surface of the feet with textured or vibratory insoles and/or open or closed-looped tactile feedback may improve gait and postural control in neurologic disorders including Parkinson’s disease (PD) [22,23,24,25,26] and multiple sclerosis [24,27] and in the elderly with cognitive impairment [28]. DT training programs also have been shown to improve gait and balance in neurodegenerative disorders such as PD [29].

We hypothesized that DT and other challenging balance tasks have the potential to reveal subtle balance impairments in “asymptomatic” *FMR1* carriers. Prior studies suggest that women PMCs asymptomatic for FXTAS display greater postural sway and poorer gait performance under DT conditions [30,31]. We previously found that PMCs without FXTAS demonstrated significantly disrupted sensory weighting for balance control such that postural sway increased when presented with conflicting visual information [32]. They also had significantly delayed response latencies to balance perturbations than controls, and these responses were not different from PMCs with FXTAS. Wearable inertial sensors are increasingly being used in neurodegenerative movement disorders, including cerebellar ataxias, to both detect prodromal signs and provide objective measures of balance and gait abnormalities because of their relatively low cost, ease of application, and provision of highly quantitative outcomes of motion impairment [20]. However, they have not been used to potentially detect prodromal postural sway deficits in asymptomatic *FMR1* PMCs or to quantify the precise postural sway deficits in patients with FXTAS. Such biomarkers of cerebellar-related balance impairments are important for the design of disease-modifying treatment trials, including rehabilitative ones in FXTAS. Thus, the aim of this study was to determine the effects of dual-tasking and other challenging balance conditions, including removing vision, reducing proprioceptive input, and narrowing the base of support on balance in both asymptomatic PMCs without FXTAS and PMCs with FXTAS using an inertial sensor system.

## 2. Materials and Methods

### 2.1. Participants

Premutation carriers (PMCs) were recruited from the Fragile X-Associated Disorders Program at Rush University Medical Center (RUMC). A detailed medical history, neurological examination, and MRI (when available) were used to diagnose FXTAS according to clinical and radiological criteria [11,12,33]. Inclusion criteria for FXTAS participants were a confirmed *FMR1* gene test indicating 55–200 CGG repeats, a diagnosis of probable or definite FXTAS, cerebellar gait ataxia on the neurological exam, the ability to stand unsupported for at least two minutes, and an age of 50 years or older. A diagnosis of probable FXTAS requires two major clinical signs (tremor and ataxia) OR one minor clinical sign (parkinsonism, peripheral neuropathy, moderate to severe short-term memory deficit, or executive function deficit) with one major radiological sign (MRI white matter lesions in the middle cerebellar peduncle or splenium of the corpus callosum). A diagnosis of definite FXTAS requires one major radiological sign and one major clinical sign. We did not include participants with possible FXTAS because these individuals might only have signs of tremor and not ataxia, and we wanted to capture the postural sway patterns in those with cerebellar ataxia. Inclusion criteria for PMCs without FXTAS were: (1) an *FMR1* gene test documenting a CGG repeat size of 55–200 and (2) a normal neurological examination with a diagnosis of no FXTAS as determined by a movement disorder expert (DAH). Inclusion criteria for controls were (1) a normal neurological examination and (2) an *FMR1* gene test showing alleles with CGG repeats < 55. Exclusion criteria for participants with FXTAS were (1) spine or lower extremity orthopedic surgery within the past year, (2) any additional neurological or musculoskeletal disorder that could potentially cause gait and balance problems, (3) history of significant head trauma, or (4) inability to follow directions for the testing protocol. Exclusion criteria for controls and PMCs without FXTAS were the same as for the participants with FXTAS but also included a significant history of tremors, balance problems, or falls. All participants signed an informed consent approved by the Institutional Review Board at RUMC. Many of the PMCs with and without FXTAS and control participants were included in previously published manuscripts including the following: (1) a DT and fast-paced gait study in FXTAS [20] and (2) a tremorography study in PMC with and without FXTAS [15].

### 2.2. Balance Testing

Quantitative balance analysis was performed using the reliable and validated APDM Mobility Lab™ instrumented postural sway (i-SWAY) inertial sensor system (APDM^TM^; Portland, OR, USA) as previously described [34,35]. An Opal™ wearable sensor was placed on the lumbar trunk (at L5, the approximate center of mass location), and sensor data were streamed wirelessly to a laptop during balance testing, with balance metrics generated by Mobility Lab 1 software (version1.3.201.v20161025-1711). Participants were asked to stand still with their arms at their sides with feet a set distance apart for the feet apart (FA) conditions. This heel-to-heel distance was scaled to their height according to that used in the Neurocom® Smart Balance Master system (NeuroCom® International, Inc.; Clackamas, OR, USA), another gold standard balance measurement system [36,37]. Different balance conditions included stance under increasingly challenging conditions. These conditions included standing with feet apart (FA)/feet together (FT) with eyes open (EO)/eyes closed (EC) on a firm and foam support surface and tandem stance (EO/EC). The foam pad was a Balance-pad Elite (Airex Balance Pad, AIREX AG, Sins, Switzerland). The ST, feet apart conditions were based on the Modified Clinical Test of Sensory Integration in Balance (mCTSIB), which is used to detect abnormal sensory–motor integration for postural control [38]. Other groups, including ours, have used similar protocols to analyze postural control under conditions of altered sensory input and narrowing the base of support while measuring postural sway with wearable inertial sensors in patients with movement disorders [34,39,40]. Participants also performed four trials in firm stance conditions under a DT condition. The DT consisted of the Controlled Oral Word Association Test (COWAT), a verbal fluency task requiring participants to name as many words as possible that begin with specific letters. The order of the conditions was from least to most difficult to allow participants with FXTAS with greater balance impairments to participate in the study. During the EO conditions, the participants were asked to look at a large X placed on a wall at eye level. All trials were 30 s in duration. We excluded data from those participants who performed the trial but were not able to maintain balance for the entire 30 s. Participants were carefully monitored during all trials for safety by a study investigator who stood directly next to the participant during the entire testing protocol. Postural sway outcomes selected for analyses were (1) 95% ellipse total sway area (TSA; m^2^/s^4^), (2) root mean square (RMS) sway (m/s^2^), and (3) jerk (m^2^/s^5^). These measures were values representing sway in both the medial–lateral and anterior–posterior directions. The 95% ellipse of the total sway area refers to the area of an ellipse covering 95% of the points in both the coronal and sagittal planes, putting more weight on regions more frequently visited. This variable excludes extreme scores outside the 95% total sway area to avoid excessive scores from skewing the results. RMS sway is the extent of postural sway calculated as the RMS of the sway angle in both the AP and ML directions and is a measure of variability in postural sway. Jerk is a measure to quantify the amount of active postural corrections or jerkiness of the sway path. Higher scores in all three variables indicate a worse balance function. These were selected a priori (out of 54 APDM-generated postural sway variables) because (1) they have good to excellent validity and reliability [35,41,42] and (2) they are sensitive measures of balance pathology in other neurological disorders, including other degenerative cerebellar ataxias [39] and PD [41,42,43,44], and were expected to be most aberrant in participants with FXTAS.

A baseline COWAT (both 30 and 60 s durations) was also performed while seated, and this was randomized to be conducted either before or after the balance tests. Participants were not told to prioritize the cognitive or motor task during the DT balance conditions. The dual-task cost (DTC %) for postural sway parameters was calculated as (DT value − ST value/ST value × 100) to assess the impact of DT cognitive interference on balance. The DTC for the verbal fluency task was calculated in the same manner using the number of correct responses given during the DT conditions on the i-SWAY as a percentage of those given while seated. Not all participants had a 30 s COWAT performed while seated because this was added to the protocol after this study began.

### 2.3. Clinical Assessments

The original FXTAS Rating Scale (FXTAS-RS) and a neurological assessment were administered by a movement disorders specialist to screen for signs and severity of FXTAS. The FXTAS-RS is a validated scale for evaluating motor symptoms and severity in FXTAS [45,46]. A FXTAS diagnosis was made by a movement disorder neurologist (DAH) according to widely used criteria [45]. The Montreal Cognitive Assessment (MoCA) test was administered to assess global cognitive function and screen for dementia [47,48,49]. Participants were asked to self-report the number of falls they had in the past 12 months. The mobility subscale of the Functional Independence Measure (FIM) [50] and the Berg Balance Scale [51] were also administered to assess activities of daily living and the level of mobility disability and functional performance-based balance levels, respectively. The Activities-Specific Balance Confidence (ABC) Scale, a self-report questionnaire rating balance confidence for performing functional balance activities in the home and community [52], was completed by all participants.

### 2.4. Molecular Analysis

Blood or buccal swab samples were sent to the Rush University Molecular Diagnostic Laboratory (Dr. Berry-Kravis’ lab) for testing *FMR1* CGG repeat size and activation ratio (AR; the fraction of cells in which the normal allele is on the active X chromosome in PMC women) testing as previously described [32,53]. These analyses were performed because CGG repeat size has been shown to have a moderating effect on disease severity in FXTAS [13,14,15], and we previously found that increased CGG repeat size in men and women with FXTAS, and reduced X activation of the normal *FMR1* allele in women, predicted balance dysfunction in PMC women with and without FXTAS [32].

### 2.5. Statistical Approach

Demographics and clinical assessment measures were compared between our three participant groups, including healthy controls, *FMR1* PMCs without FXTAS (no FXTAS group), and *FMR1* PMCs with probable or definite FXTAS (FXTAS group), with a one-way ANOVA followed by the Tukey’s post hoc comparisons test (for normally distributed, parametric variables) or the Kruskal–Wallis test with the Dunn post hoc comparisons test (for nonnormal, nonparametric variables). Correlations between i-SWAY measures and the MoCA, BMI, CGG repeat size, AR (women), FXTAS-RS scores, ABC, and BBS, were examined in the FXTAS group using Spearman’s rho at the *p* = 0.05 significance level. Sex differences in demographic and clinical characteristics and postural sway variables within each group were examined via Student’s *t*-tests or Mann–Whitney U tests depending on the normality of the data.

All postural sway parameters were first screened for significance between controls, PMCs without FXTAS (no FXTAS), and those with FXTAS using the Kruskal–Wallis test with the Dwass, Steel, Critchlow-Fligner (DSCF) multiple comparison analysis as the post hoc test. False Discovery Rate (FDR) corrections using the two-stage linear step-up procedure of Benjamini, Krieger, and Yekutieli [54] were applied to avoid a Type-I error because of the large number of conditions and outcomes tested. Significant findings from this analysis were then entered into a multiple regression model controlling for age and MoCA scores because these were significantly different between groups and thought to be potential confounders. Sex was not included in the regression model because we first examined if there were sex differences in the postural sway outcomes in each of the 3 groups, and there were only a few significant differences out of the 48 variables tested in the FXTAS and no FXTAS groups. CGG repeat size and AR were also not placed in the final model because repeat number did not correlate with any postural sway parameter under any condition and AR correlated weakly with only a few parameters in the PMC groups, none of which were found to be significantly different from the controls. BMI was not entered into the regression model because it did not correlate with any postural sway parameters.

To determine the effect of sensory manipulation on postural sway, differences among the 3 groups in the conditions of the mCTSIB were examined by calculating the postural change scores in TSA, jerk, and RMS sway relative to the baseline FAEO ST, firm condition and the (1) FAEC ST, firm, (2) FAEO ST, foam, and (3) FAEC ST, foam conditions and by performing the Kruskal–Wallis test with the Dunn post hoc test for multiple comparison analyses.

Spearman’s rank correlation coefficients (*rho*) with FDR corrections were used to assess the relationship between the i-SWAY parameters and the FXTAS-RS, BBS, FIM, and ABC scores. All statistical analyses were performed in Graph Pad Prism (version 10.0) except for the regression analysis, which was performed using SAS 9.4 (SAS Institute Inc., Cary, NC, USA) at the 5% significance level.

For comparisons in COWAT performance among the 3 groups during ST (while seated) and DT (while balancing) and any DTC (calculated as DT-ST/ST × 100), we performed one-way ANOVAs followed by the Tukey’s post hoc comparisons test (for normally distributed variables) or the Kruskal–Wallis test with the Dunn post hoc comparisons test (for non-normally distributed variables).

## 3. Results

### 3.1. Participant Characteristics

The demographic and clinical characteristics of the study participants are shown in Table 1. Thirty-three individuals with FXTAS (68.6 ± 8.3 years), 34 PMCs without FXTAS (54.9 ± 9.5 years), and 48 healthy controls (64.0 + 10.5 years) participated in this study. PMCs without FXTAS were significantly younger than those with FXTAS and controls. MoCA scores were significantly lower in the FXTAS group compared with those without FXTAS, and men with FXTAS scored significantly lower than women on the MoCA (*p* = 0.0012). Using a cut-off score ≤ 25 [49], thirteen individuals with FXTAS had mild cognitive impairment and all but two of these were men. BMI was significantly greater in the FXTAS group compared with those without FXTAS and the controls. Years of education were significantly lower in the FXTAS group compared with the controls. As expected, FIM, BBS, and ABC scores were significantly lower, and falls were more frequent in participants with FXTAS than in the controls and PMCs without FXTAS.

### 3.2. Postural Sway Results

Of the 42 total balance conditions tested, 36 revealed significant differences between groups after applying FDR corrections. These 36 postural sway variables were then included in a linear regression analysis model controlling for age and MoCA scores. Age was included because the no FXTAS group was significantly younger than the control and FXTAS groups and the MoCA was included because MoCA scores were significantly lower in the FXTAS and no FXTAS groups, and both could have been potential confounders. In addition, age and MoCA scores were significantly correlated with many postural sway parameters.

**Figure 1:**
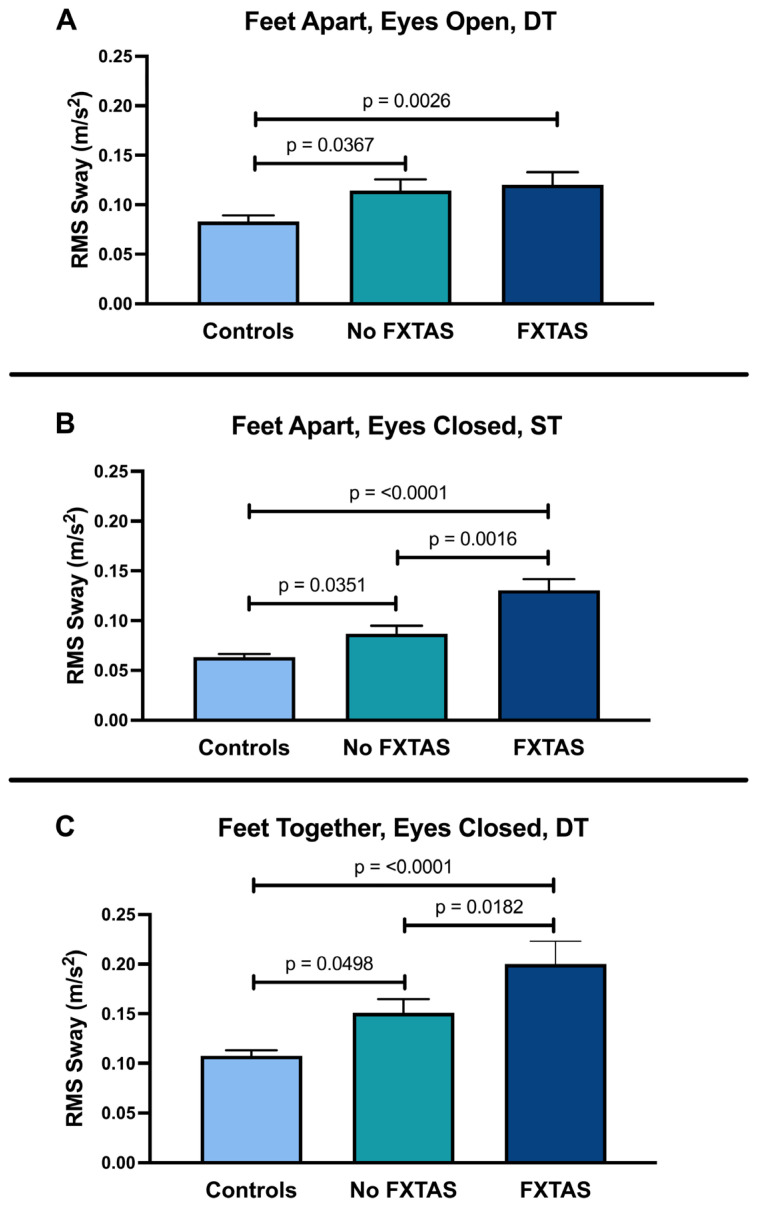
Mean postural sway data during 30 s i-SWAY tests completed by individuals with FXTAS, PMCs without FXTAS (No FXTAS), and healthy controls. Significant differences between the no FXTAS group and controls were elicited under the following conditions: (**A**) feet apart, eyes open, dual-tasking, firm surface (FAEO DT, firm), (**B**) feet apart, eyes closed, single-tasking, firm surface (FAEC ST, firm), and (**C**) feet together, eyes closed, dual-tasking, firm surface (FTEC DT, firm). All data are reported as mean ± SEM.

**Figure 2:**
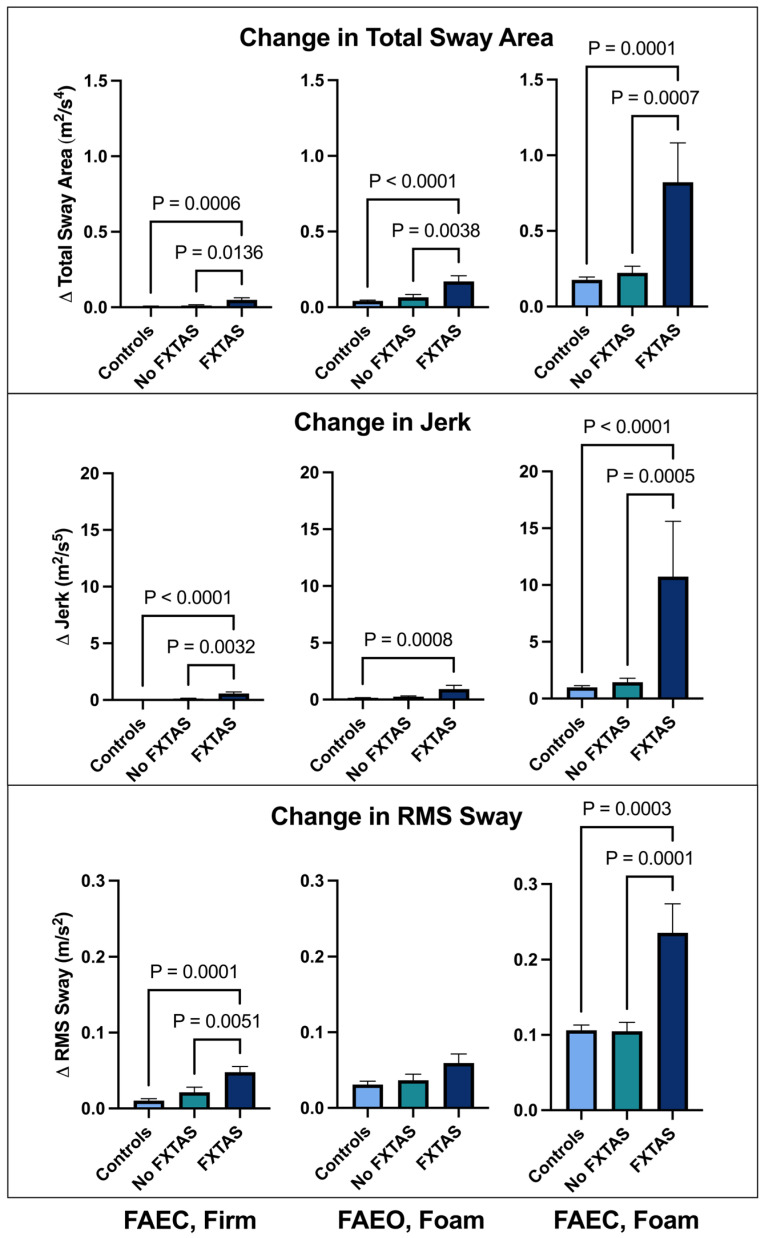
Change in postural sway following sensory manipulation using the Modified Clinical Test for Sensory Integration and Balance (mCTSIB) in individuals with FXTAS, PMCs without FXTAS (no FXTAS), and healthy controls. All change scores are relative to the feet apart, eyes open, firm surface (FAEO, firm) condition. All data are reported as mean ± SEM. FAEC—firm, feet apart, eyes closed, firm surface; FAEO—foam, feet apart, eyes open, foam surface; FAEC—foam, feet apart, eyes closed, foam surface.

Postural sway outcomes and the results of the regression analysis are shown in Table 2. The FXTAS group performed significantly worse than both the controls and the no FXTAS group in almost all balance conditions. The no FXTAS group performed similarly to the healthy controls in most of the balance conditions except for three postural sway parameters that were significantly worse than controls. First, when participants were asked to stand on a firm surface with their feet apart (FA) and eyes open (EO) while simultaneously performing the cognitive task (FAEO DT, firm condition), RMS sway was found to be significantly greater in both the no FXTAS group (*p* = 0.0367) and the FXTAS group (*p* = 0.0026) compared with the controls (Table 2; Figure 1A). RMS sway was also significantly increased in the no FXTAS group compared with the controls in the (1) firm surface, feet apart, and eyes closed ST condition (FAEC ST, firm; *p* = 0.0351; Figure 1B) and (2) firm surface, feet together, and eyes closed while simultaneously performing the cognitive dual task (FTEC DT, firm; *p* = 0.0498; Figure 1C). Thus, a measurable impairment in RMS sway, signifying greater postural sway variability, was found in the no FXTAS group compared with the healthy controls. A statistical trend (*p* < 0.10) was found for higher RMS sway in the no FXTAS group than controls in the following five additional conditions: (1) FTEO ST, firm (*p* = 0.0795), (2) FTEC ST, firm (*p* = 0.0706, (3) FAEC DT, firm (*p* = 0.076), (4) FTEO DT, firm (*p* = 0.0682), and (5) FTEO, foam (*p* = 0.0537). There were two postural sway conditions that revealed significant differences between the controls and FXTAS groups but were not different between the FXTAS and no FXTAS groups (1: TSA and RMS sway in the FAEO DT, firm condition, 2: RMS sway in the FTEO DT, firm condition).

The dual-task cost data are listed in Table 3. There were no significant differences in DTC for postural sway variables among the controls, PMCs without FXTAS, and those with FXTAS except for a lower DTC for jerk in the FTEC, firm condition in participants with FXTAS compared to control participants (*p* = 0.0357).

Changes in postural sway scores on the conditions of the mCTSIB relative to baseline are shown in Figure 2. The FXTAS group demonstrated a significantly increased change in TSA, jerk, and RMS sway compared with the controls and PMCs without FXTAS under both eyes closed conditions (FAEC ST, firm and FAEC ST, foam). Under the FAEO ST, foam condition, the FXTAS group exhibited significantly increased TSA compared with the control and no FXTAS groups, and significantly greater jerk than controls. There were no significant differences between the control, no FXTAS, and FXTAS groups in the RMS sway domain under FAEO ST, foam stance.

### 3.3. Cognitive Test Results

The FXTAS group had significantly lower performance on the COWAT during the DT balance conditions than the controls and PMCs without FXTAS, but there were no differences in DTC for performance on the COWAT in the participants with FXTAS compared to the controls (Table 4). The asymptomatic PMCs without FXTAS performed significantly better than the participants with FXTAS on all DT COWAT conditions and one ST COWAT condition.

### 3.4. Correlational Analyses

Correlations between all postural sway parameters and FXTAS-RS, ABC, BBS, and FIM mobility scores, in the FXTAS group only are shown in Table 5. FXTAS-RS scores correlated positively with 26 postural sway measures. The ABC correlated negatively with 36 postural sway parameters. The BBC correlated with six postural sway parameters, and the FIM mobility subscale did not correlate with any postural sway measures.

## 4. Discussion

The most novel finding of this study was that postural sway deficits in the RMS postural sway variability domain under EC and DT conditions were found in asymptomatic PMCs and that these deficits might represent prodromal signs of FXTAS. This is entirely plausible as RMS in postural sway represents variability in the sway acceleration path, and movement timing variability is a main feature of cerebellar ataxia [55,56,57]. Furthermore, there was no significant difference in RMS sway between the no FXTAS and FXTAS groups in the FAEO DT condition, suggesting that this specific balance outcome is consistent with the FXTAS phenotype. Interestingly, increased RMS sway in the PMCs without FXTAS group compared with the controls reached statistical trends for five other balance conditions, lending support for this hypothesis. Our findings are somewhat similar to that of another group that investigated postural sway in individuals who were presymptomatic for Spinocerebellar Ataxia Type 2 (SCA 2) [58]. However, in that study, increased RMS sway, jerk, and path length were found in tandem stance (which was the only stance position examined).

Surprisingly, we did not observe significant differences in TSA, RMS sway, or jerk between the control, no FXTAS, and FXTAS groups in the FTEC foam condition and the tandem EO condition, which are very difficult for persons with cerebellar ataxia and balance dysfunction to perform. The small number of participants with FXTAS that could perform the FTEC foam task for 30 s (n = 7) likely was related to this lack of significance. The mean postural sway scores during the tandem EO condition were much higher in FXTAS participants than in the controls, but the range and standard deviations of the scores were high, so statistical significance was not achieved. We also anticipated that postural sway would be significantly worse in tandem stance in the no FXTAS group compared with the controls, but this was not the case. These findings were unexpected because difficulty with tandem standing and especially tandem gait are hallmark features of cerebellar ataxia. Perhaps the higher variability in the no FXTAS group compared with the controls accounted for the lack of significance in the tandem stance conditions. Another confounding factor is that individuals who could not perform the task for 30 s were excluded from the analyses because the results would not be comparable in persons who were losing their balance or falling. This resulted in the exclusion of data from 5 PMCs without FXTAS in the FTEC foam condition and 10 in the tandem EO condition. It is feasible that postural sway during tandem stance might not serve as a good biomarker for predicting FXTAS, but future studies with equal numbers of men and women PMCs without FXTAS are needed to further explore this possibility. The group that examined tandem standing and walking in prodromal SCA-2 using inertial sensors found excessive trunk ROM compared with controls during tandem gait [58], suggesting this might also be a good digital biomarker for genetic neurodegenerative ataxias like FXTAS. We plan to further investigate this possibility in future work.

The two postural sway conditions that revealed significantly worse postural sway between the controls and FXTAS groups, which were not different between the FXTAS and no FXTAS groups (increased TSA in the FAEO DT condition and increased RMS sway in the FTEO DT condition), suggest that the asymptomatic PMCs were developing the FXTAS phenotype. However, we cannot conclude this decisively because there were only statistical trends for differences between the controls and asymptomatic PMCs for postural sway in these conditions.

We hypothesized that DTC for postural sway would be significantly higher in the FXTAS and perhaps no FXTAS groups because dual-tasking was expected to increase the neural requirements for balance control, thereby causing DT cognitive–motor interference, but this was not found in the present study. This is similar to our prior gait findings, where there was no DTC for spatiotemporal aspects of straight-line gait in FXTAS, although there was significant DTC for turning speed in men with FXTAS [20]. In our present study, we in fact found that DTC for jerk was significantly lower in FXTAS than the controls in the FTEC DT, firm condition. One possible explanation for these findings is that the individuals with FXTAS prioritized balance over cognitive performance during DT balance testing. This is in line with our findings of significantly lower performance on the COWAT during the DT balance conditions in the participants with FXTAS than in the controls and PMCs without FXTAS. However, there were no differences in DTC for performance on the COWAT between the groups. This is likely due to the finding that ST performance on the COWAT was very similar to DT performance while balancing, suggesting a ceiling in performance on this cognitive task was reached during ST seating.

We sought to evaluate how sensory input impacts postural sway for all three groups. The change in postural sway scores revealed that the participants with FXTAS exhibited significantly increased TSA, jerk, and RMS sway compared with both the control and no FXTAS group under the FAEC ST, firm and foam conditions. However, there were no results indicating worsened postural sway during sensory manipulation in PMCs without FXTAS compared to the controls. Taken together, these results indicate that the participants with FXTAS rely on visual input to maintain a stable balance more than the controls and PMCs without FXTAS and the reduction in proprioceptive input combined with removing visual input increases postural sway the most. These findings are similar to those we previously reported in FXTAS using the Neurocom Balance Master system, where the vestibular control of balance was most impaired [32]. This result is expected given the crucial role of the cerebellum in the vestibular control of balance.

Our finding that the FXTAS-RS scores correlated with 29 postural sway parameters in the participants with FXTAS indicates that these measures relate to the FXTAS phenotype and motor disease severity and may potentially be good outcome measures of balance function in future FXTAS studies, including clinical trials. This will require future studies using machine learning approaches to narrow down the list of pertinent balance outcome measures that distinguish FXTAS from controls and that correlate with disease severity.

The limitations of this study include the low numbers of PMC men without FXTAS in this group. It is very difficult to find and enroll men without FXTAS into FXTAS studies because the most likely way to recruit and find PMCs without FXTAS is when parents present with their children with FXS to a larger FXS clinic (like ours at RUMC run by EBK) and women are obligate PMCs. Efforts to obtain PMC carrier men into prodromal and longitudinal studies will likely be facilitated by the creation of registries like the recently established International Fragile X Premutation Registry [59]. The limitations also include having to exclude data from participants who could not perform the trial for the entire 30 s. Future studies exploring shorter trial durations may be worthwhile.

Our future work is designed to follow PMCs with abnormal postural sway scores in the RMS domain to ascertain whether they phenoconvert to FXTAS. In addition, we aim to determine the best balance and gait digital outcome measures that (1) distinguish individuals with FXTAS from healthy controls, (2) are highly associated with disease severity and progression, and (3) are useful for fall risk prediction.

## 5. Conclusions

We identified three postural sway conditions (FAEO DT, firm; FAEC ST, firm; and FTEC DT, firm) that elicited significant RMS sway deficits in PMCs without symptoms of FXTAS. These findings may represent prodromal signs of FXTAS that could potentially serve as sensitive digital biomarkers of FXTAS onset and should be explored in future clinical and rehabilitation studies. Additionally, individuals with FXTAS demonstrated the most severe increases in postural sway relative to the controls and PMCs without FXTAS when vision was removed and proprioception was reduced, indicating poor vestibular control of balance.

## Figures and Tables

**Table 1:** Participant characteristics.

Variable	Controls (*n* = 48)	No FXTAS (*n* = 34)	FXTAS (*n* = 33)	ANOVA *p*-Value	Controls vs. No FXTAS	Controls vs. FXTAS	No FXTAS vs. FXTAS
Age	64.00 ± 10.45	54.94 ± 9.51	68.55 ± 8.31	***p* ≤ 0.0001**	***p* = 0.0002**	*p* = 0.0959	***p* ≤ 0.0001**
Sex (*n*, %)							
Men	22 (45.83%)	4 (11.76%)	21 (63.64%)				
Women	26 (54.17%)	30 (88.24%)	12 (36.36%)				
Height (cm)	169.82 ± 9.39	166.38 ± 7.35	170.40 ± 11.14	*p* = 0.1769			
Weight (kg)	76.58 ± 15.24	71.81 ± 20.52	91.00 ± 29.54	***p* = 0.0014**	*p* = 0.2382	*p* = 0.0918	***p* = 0.0009**
BMI	26.43 ± 4.15	25.68 ± 5.70	31.15 ± 8.98	***p* = 0.0026**	*p* = 0.6954	***p* = 0.0424**	***p* = 0.0023**
Years of Education	18.51 ± 3.21	17.03 ± 2.97	15.76 ± 2.40	***p* = 0.0013**	*p* = 0.1196	***p* = 0.0010**	*p* = 0.4492
FXTAS Diagnosis (*n*, %)							
Probable	N/A	N/A	21 (63.64%)				
Definite	N/A	N/A	12 (36.36%)				
*FMR1* CGG Repeats	30.62 ± 4.67	92.14 ± 20.29	86.58 ± 20.19	***p* ≤ 0.0001**	***p* ≤ 0.0001**	***p* ≤ 0.0001**	*p* ≥ 0.9999
Activation Ratios (Women)	N/A	44.81 ± 21.36	44.13 ± 18.15	*p* = 0.9368			
FXTAS-RS	10.55 ± 7.02	6.56 ± 3.43	34.69 ± 24.11	***p* ≤ 0.0001**	*p* = 0.2057	***p* ≤ 0.0001**	***p* ≤ 0.0001**
BBS	55.66 ± 0.81	55.82 ± 0.72	50.74 ± 4.70	***p* ≤ 0.0001**	*p* ≥ 0.9999	***p* ≤ 0.0001**	***p* ≤ 0.0001**
FIM Mobility Subscale	35.00 ± 0.00	34.91 ± 0.29	34.10 ± 1.21	***p* ≤ 0.0001**	*p* = 0.9909	***p* ≤ 0.0001**	***p* ≤ 0.0001**
ABC (%)	96.18 ± 4.40	94.44 ± 5.15	76.82 ± 16.79	***p* ≤ 0.0001**	*p* = 0.4381	***p* ≤ 0.0001**	***p* ≤ 0.0001**
1 Year Retrospective Falls (#)	0.29 ± 0.59	0.84 ± 1.39	3.13 ± 7.88	***p* = 0.0012**	*p* = 0.1679	***p* = 0.0008**	*p* = 0.3008
MoCA	27.48 ± 2.14	27.85 ± 1.94	26.21 ± 2.68	***p* = 0.0241**	*p* ≥ 0.9999	*p* = 0.1163	***p* = 0.0264**
COWAT SS	103.25 ± 13.52	103.56 ± 13.87	92.42 + 12.55	***p* = 0.0011**	*p* ≥ 0.9999	***p* = 0.0016**	***p* = 0.0077**

Data reported as mean ± SD, unless otherwise noted. Any significant comparisons (*p* < 0.05) are bolded. BMI, Body Mass Index; FXTAS-RS, FXTAS Rating Scale; BBS, Berg Balance Scale; FIM, Functional Independence Measure; ABC, Activities-specific Balance Confidence Scale; MoCA, Montreal Cognitive Assessment; COWAT SS, Controlled Oral Word Association Test standard score, scaled to age and years of education.

**Table 2:** Postural sway comparisons among the controls, PMCs without FXTAS (no FXTAS), and PMCs with FXTAS.

Variable	Controls	No FXTAS	FXTAS	Controls vs. No FXTAS	Controls vs. FXTAS	No FXTAS vs. FXTAS
Condition: Feet apart, eyes open, firm surface, ST						
	*n* = 48	*n* = 34	*n* = 33			
Total Sway Area (m^2^/s^4^)	0.02 ± 0.01	0.03 ± 0.02	0.05 ± 0.07	*p* = 0.3379	***p* ≤ 0.0001**	***p* = 0.0112**
Jerk (m^2^/s^5^)	0.09 ± 0.09	0.09 ± 0.07	0.38 ± 0.61	*p* = 0.7571	***p* = 0.0005**	***p* = 0.0013**
RMS Sway (m/s^2^)	0.05 ± 0.02	0.07 ± 0.03	0.09 ± 0.07	*p* = 0.2913	***p* ≤ 0.0001**	***p* = 0.0142**
Condition: Feet apart, eyes closed, firm surface, ST						
	*n* = 48	*n* = 34	*n* = 32			
Total Sway Area (m^2^/s^4^)	0.02 ± 0.02	0.04 ± 0.04	0.10 ± 0.12	*p* = 0.2522	***p* ≤ 0.0001**	***p* = 0.0064**
Jerk (m^2^/s^5^)	0.14 ± 0.11	0.22 ± 0.22	0.95 ± 1.18	*p* = 0.6315	***p* ≤ 0.0001**	***p* = 0.0002**
RMS Sway (m/s^2^)	0.06 ± 0.02	0.09 ± 0.05	0.13 ± 0.06	***p* = 0.0351**	***p* ≤ 0.0001**	***p* = 0.0016**
Condition: Feet together, eyes open, firm surface, ST						
	*n* = 48	*n* = 34	*n* = 33			
Total Sway Area (m^2^/s^4^)	0.06 ± 0.04	0.10 ± 0.11	0.19 ± 0.16	*p* = 0.1712	***p* ≤ 0.0001**	***p* = 0.0012**
Jerk (m^2^/s^5^)	0.19 ± 0.13	0.27 ± 0.19	0.88 ± 1.81	*p* = 0.9216	***p* = 0.0029**	***p* = 0.0096**
RMS Sway (m/s^2^)	0.08 ± 0.03	0.11 ± 0.06	0.15 ± 0.07	*p* = 0.0795 †	***p* ≤ 0.0001**	***p* = 0.0029**
Condition: Feet together, eyes closed, firm surface, ST						
	*n* = 48	*n* = 34	*n* = 24			
Total Sway Area (m^2^/s^4^)	0.12 ± 0.13	0.19 ± 0.18	0.54 ± 0.68	*p* = 0.4999	***p* ≤ 0.0001**	***p* = 0.0008**
Jerk (m^2^/s^5^)	0.48 ± 0.67	0.61 ± 0.48	5.25 ± 17.17	*p* = 0.6903	***p* = 0.0232**	***p* = 0.0233**
RMS Sway (m/s^2^)	0.11 ± 0.05	0.14 ± 0.06	0.22 ± 0.13	*p* = 0.0706 †	***p* ≤ 0.0001**	***p* = 0.0009**
Condition: Feet apart, eyes open, firm surface, DT (COWAT letter C)						
	*n* = 48	*n* = 34	*n* = 33			
Total Sway Area (m^2^/s^4^)	0.05 ± 0.05	0.07 ± 0.07	0.08 ± 0.10	*p* = 0.0934 †	***p* = 0.0207**	*p* = 0.6162
Jerk (m^2^/s^5^)	0.40 ± 0.47	0.45 ± 0.49	0.72 ± 1.21	N/A	N/A	N/A
RMS Sway (m/s^2^)	0.08 ± 0.04	0.11 ± 0.07	0.12 ± 0.07	***p* = 0.0367**	***p* = 0.0026**	*p* = 0.4545
Condition: Feet apart, eyes closed, firm surface, DT (COWAT letter L)						
	*n* = 48	*n* = 34	*n* = 33			
Total Sway Area (m^2^/s^4^)	0.04 ± 0.06	0.07 ± 0.08	0.11 ± 0.11	*p* = 0.1523	***p* ≤ 0.0001**	***p* = 0.0255**
Jerk (m^2^/s^5^)	0.37 ± 0.44	0.38 ± 0.44	1.23 ± 1.74	*p* = 0.7156	***p* = 0.0007**	***p* = 0.0105**
RMS Sway (m/s^2^)	0.08 ± 0.04	0.11 ± 0.07	0.14 ± 0.08	*p* = 0.0760 †	***p* ≤ 0.0001**	***p* = 0.0165**
Condition: Feet together, eyes open, firm surface, DT (COWAT letter A)						
	*n* = 48	*n* = 34	*n* = 33			
Total Sway Area (m^2^/s^4^)	0.09 ± 0.08	0.17 ± 0.13	0.31 ± 0.53	*p* = 0.4273	***p* = 0.0011**	***p* = 0.0374**
Jerk (m^2^/s^5^)	0.47 ± 0.34	0.67 ± 0.73	1.56 ± 4.22	N/A	N/A	N/A
RMS Sway (m/s^2^)	0.10 ± 0.04	0.15 ± 0.10	0.18 ± 0.15	*p* = 0.0682 †	***p* = 0.0002**	*p* = 0.1077
Condition: Feet together, eyes closed, firm surface, DT (COWAT letter S)						
	*n* = 47	*n* = 34	*n* = 27			
Total Sway Area (m^2^/s^4^)	0.11 ± 0.05	0.20 ± 0.15	0.42 ± 0.52	*p* = 0.3706	***p* ≤ 0.0001**	***p* = 0.0020**
Jerk (m^2^/s^5^)	0.63 ± 0.42	0.76 ± 0.59	2.92 ± 5.92	*p* = 0.7917	***p* = 0.0019**	***p* = 0.0043**
RMS Sway (m/s^2^)	0.11 ± 0.04	0.15 ± 0.08	0.20 ± 0.12	***p* = 0.0498**	***p* ≤ 0.0001**	***p* = 0.0182**
Condition: Feet apart, eyes open, foam						
	*n* = 48	*n* = 34	*n* = 33			
Total Sway Area (m^2^/s^4^)	0.06 ± 0.04	0.09 ± 0.12	0.22 ± 0.26	*p* = 0.4412	***p* ≤ 0.0001**	***p* = 0.0022**
Jerk (m^2^/s^5^)	0.25 ± 0.22	0.35 ± 0.43	1.31 ± 2.10	*p* = 0.6644	***p* = 0.0005**	***p* = 0.0099**
RMS Sway (m/s^2^)	0.08 ± 0.03	0.10 ± 0.05	0.15 ± 0.08	*p* = 0.2698	***p* ≤ 0.0001**	***p* = 0.0013**
Condition: Feet apart, eyes closed, foam						
	*n* = 48	*n* = 34	*n* = 24			
Total Sway Area (m^2^/s^4^)	0.19 ± 0.13	0.25 ± 0.26	0.85 ± 1.28	*p* = 0.9415	***p* = 0.0002**	***p* = 0.0012**
Jerk (m^2^/s^5^)	1.09 ± 1.05	1.53 ± 2.08	10.98 ± 24.26	*p* = 0.6261	***p* = 0.0012**	***p* = 0.0018**
RMS Sway (m/s^2^)	0.16 ± 0.06	0.17 ± 0.07	0.31 ± 0.20	*p* = 0.7158	***p* ≤ 0.0001**	***p* = 0.0003**
Condition: Feet together, eyes open, foam						
	*n* = 48	*n* = 34	*n* = 28			
Total Sway Area (m^2^/s^4^)	0.13 ± 0.09	0.19 ± 0.14	0.50 ± 0.49	*p* = 0.1882	***p* ≤ 0.0001**	***p* = 0.0007**
Jerk (m^2^/s^5^)	0.75 ± 0.56	1.00 ± 1.05	2.78 ± 3.13	*p* = 0.3010	***p* ≤ 0.0001**	***p* = 0.0033**
RMS Sway (m/s^2^)	0.12 ± 0.04	0.14 ± 0.05	0.22 ± 0.12	*p* = 0.0537 †	***p* ≤ 0.0001**	***p* = 0.0009**
Condition: Feet together, eyes closed, foam						
	*n* = 48	*n* = 29	*n* = 7			
Total Sway Area (m^2^/s^4^)	0.77 ± 0.49	0.95 ± 0.63	0.74 ± 0.56	N/A	N/A	N/A
Jerk (m^2^/s^5^)	4.43 ± 3.04	4.80 ± 3.74	4.62 ± 3.77	N/A	N/A	N/A
RMS Sway (m/s^2^)	0.29 ± 0.09	0.32 ± 0.12	0.27 ± 0.11	N/A	N/A	N/A
Condition: Tandem, eyes open						
	*n* = 30	*n* = 24	*n* = 11			
Total Sway Area (m^2^/s^4^)	0.18 ± 0.25	0.40 ± 0.99	0.41 ± 0.59	N/A	N/A	N/A
Jerk (m^2^/s^5^)	2.19 ± 2.30	2.38 ± 3.80	3.88 ± 5.29	N/A	N/A	N/A
RMS Sway (m/s^2^)	0.13 ± 0.07	0.17 ± 0.16	0.19 ± 0.12	N/A	N/A	N/A
Condition: Tandem, eyes closed						
	*n* = 10	*n* = 14	*n* = 2			
Total Sway Area (m^2^/s^4^)	0.54 ± 0.72	3.43 ± 6.45		N/A	N/A	N/A
Jerk (m^2^/s^5^)	5.69 ± 6.38	17.78 ± 20.41		N/A	N/A	N/A
RMS Sway (m/s^2^)	0.22 ± 0.12	0.50 ± 0.47		N/A	N/A	N/A

Data reported as mean ± SD. Any significant comparisons (*p* < 0.05) are bolded. Comparisons that are not significantly different between the no FXTAS and FXTAS groups are italicized. † Denotes a statistical trend with *p*-values between 0.051 and 0.10. PMC, premutation carrier; ST, single task; DT, dual task. This table lists the *p*-values from the regression analysis (controlling for age and MoCA) for those variables that were found to be significant following Kruskal–Wallis tests with FDR corrections.

**Table 3:** Dual-task costs while balancing in the control, no FXTAS, and FXTAS groups.

Variable	Controls	No FXTAS	FXTAS	Controls vs. No FXTAS	Controls vs. FXTAS	No FXTAS vs. FXTAS
Condition: Feet apart, eyes open, firm surface, DTC						
	*n* = 48	*n* = 34	*n* = 33			
Total Sway Area (m^2^/s^4^)	223.42 ± 309.69	268.34 ± 424.03	127.06 ± 139.94	N/A	N/A	N/A
Jerk (m^2^/s^5^)	412.40 ± 612.27	493.31 ± 769.31	192.47 ± 247.85	*p* = 0.7185	*p* = 0.3181	*p* = 0.2522
RMS Sway (m/s^2^)	64.64 ± 94.83	87.18 ± 118.21	48.38 ± 60.17	N/A	N/A	N/A
Condition: Feet apart, eyes closed, firm surface, DTC						
	*n* = 48	*n* = 34	*n* = 32			
Total Sway Area (m^2^/s^4^)	132.60 ± 291.01	99.70 ± 178.33	43.39 ± 95.48	N/A	N/A	N/A
Jerk (m^2^/s^5^)	206.46 ± 368.49	106.34 ± 175.84	74.17 ± 269.25	*p* = 0.1041	*p* = 0.1534	*p* = 0.8442
RMS Sway (m/s^2^)	25.01 ± 56.23	27.90 ± 66.31	11.59 ± 46.04	N/A	N/A	N/A
Condition: Feet together, eyes open, firm surface, DTC						
	*n* = 48	*n* = 34	*n* = 33			
Total Sway Area (m^2^/s^4^)	91.42 ± 204.53	154.68 ± 330.03	53.76 ± 119.09	N/A	N/A	N/A
Jerk (m^2^/s^5^)	186.72 ± 181.11	246.75 ± 496.43	68.70 ± 95.54	*p* = 0.5210	*p* = 0.1711	*p* = 0.0904
RMS Sway (m/s^2^)	29.26 ± 59.13	64.30 ± 149.21	20.77 ± 59.43	N/A	N/A	N/A
Condition: Feet together, eyes closed, firm surface, DTC						
	*n* = 47	*n* = 34	*n* = 24			
Total Sway Area (m^2^/s^4^)	13.92 ± 62.32	47.04 ± 165.39	−20.87 ± 44.27	*p* = 0.3857	*p* = 0.2412	*p* = 0.0865
Jerk (m^2^/s^5^)	71.27 ± 100.36	68.38 ± 161.83	−10.24 ± 65.09	*p* = 0.6456	***p* = 0.0357**	*p* = 0.1474
RMS Sway (m/s^2^)	4.20 ± 28.48	17.63 ± 79.35	−12.82 ± 27.29	N/A	N/A	N/A

Data reported as mean ± SD. Any significant comparisons (*p* < 0.05) are bolded. DTC, dual-task cost, which was calculated using the formula (DT − ST)/ST × 100.

**Table 4:** Cognitive performance on the COWAT during single task (ST) or dual task (DT).

Variable	Controls (*n* = 17)	No FXTAS (*n* = 20)	FXTAS (*n* = 18)	ANOVA *p*-Value	Controls vs. No FXTAS	Controls vs. FXTAS	No FXTAS vs. FXTAS
COWAT “C”—ST	9.76 ± 2.75	9.70 ± 2.60	7.72 ± 2.85	***p* = 0.023**	*p* ≥ 0.999	***p* = 0.048**	*p* = 0.057
COWAT “C”—DT	10.71 ± 3.27	10.00 ± 2.83	7.61 ± 1.85	***p* = 0.003**	*p* = 0.711	***p* = 0.004**	***p* = 0.024**
COWAT “C”—DTC	16.87 ± 38.94	6.39 ± 29.79	9.94 ± 47.49	*p* = 0.716	N/A	N/A	N/A
COWAT “L”—ST	9.88 ± 2.74	9.35 ± 1.95	7.61 ± 2.52	***p* = 0.018**	*p* = 0.781	***p* = 0.020**	*p* = 0.076
COWAT “L”—DT	11.06 ± 2.61	10.50 ± 2.48	7.94 ± 1.98	***p* = 0.0005**	*p* = 0.756	***p* = 0.0009**	***p* = 0.005**
COWAT “L”—DTC	16.97 ± 32.16	13.05 ± 17.68	13.25 ± 37.66	*p* = 0.608	N/A	N/A	N/A
COWAT “A”—ST	9.35 ± 2.64	8.75 ± 1.97	6.88 ± 2.37	***p* = 0.008**	*p* = 0.713	***p* = 0.009**	***p* = 0.047**
COWAT “A”—DT	10.18 ± 3.03	9.45 ± 2.72	6.67 ± 2.22	***p* = 0.0006**	*p* = 0.690	***p* = 0.0008**	***p* = 0.006**
COWAT “A”—DTC	14.50 ± 41.65	11.53 ± 31.95	24.86 ± 6.03	*p* = 0.264	N/A	N/A	N/A
COWAT “S”—ST	10.88 ± 2.32	10.10 ± 1.89	8.59 ± 2.43	***p* = 0.012**	*p* = 0.533	***p* = 0.010**	*p* = 0.104
COWAT “S”—DT	12.18 ± 3.83	10.40 ± 2.48	7.87 ± 2.39	***p* = 0.0005**	*p* = 0.173	***p* = 0.0003**	***p* = 0.037**
COWAT “S”—DTC	13.66 ± 35.36	3.56 ± 20.98	−7.65 ± 37.16	*p* = 0.078	N/A	N/A	N/A

Data are reported as mean ± SD. Any significant comparisons (*p* < 0.05) are bolded. ST, single task while seated; DT, dual task while balancing.

**Table 5:** Spearman’s correlation coefficients (*rho*) among the i-SWAY parameters, FXTAS-RS, ABC, BBS, and FIM scores.

Variable	FXTAS-RS	ABC	BBS	FIM
Age	0.228	0.216	−0.377	−0.194
BMI	−0.342	−0.067	−0.089	0.158
Years of Education	0.011	0.136	0.143	−0.171
MoCA	−0.341	0.151	0.303	0.009
COWAT SS	−0.351	0.298	0.162	0.094
FXTAS-RS	--	−0.324	−0.425	−0.301
ABC	−0.324	--	0.524 *	0.454
BBS	−0.425 *	0.524 **	--	0.613 *
FIM	−0.301	0.454 *	0.613 *	--
1 Year Retrospective Falls	0.112	−0.438 *	−0.192	−0.228
Condition: Feet apart, eyes open, firm surface, ST				
Total Sway Area (m^2^/s^4^)	0.472 *	−0.358 *	−0.121	0.019
Jerk (m^2^/s^5^)	0.435 *	−0.469 *	−0.344	−0.182
RMS Sway (m/s^2^)	0.450 *	−0.337 *	0.026	0.133
Condition: Feet apart, eyes closed, firm surface, ST				
Total Sway Area (m^2^/s^4^)	0.460 *	−0.436 *	−0.380	−0.369
Jerk (m^2^/s^5^)	0.383	−0.420 *	−0.402	−0.378
RMS Sway (m/s^2^)	0.406 *	−0.610 **	−0.366	−0.408
Condition: Feet together, eyes open, firm surface ST				
Total Sway Area (m^2^/s^4^)	0.431 *	−0.618 **	−0.393	−0.371
Jerk (m^2^/s^5^)	0.646 **	−0.532 **	−0.448	−0.436
RMS Sway (m/s^2^)	0.356	−0.617 **	−0.311	−0.333
Condition: Feet together, eyes closed, firm surface, ST				
Total Sway Area (m^2^/s^4^)	0.433	−0.653 **	−0.466	−0.163
Jerk (m^2^/s^5^)	0.539 *	−0.491 *	−0.454	−0.077
RMS Sway (m/s^2^)	0.437	−0.642 **	−0.446	−0.178
Condition: Feet apart, eyes open, firm surface, DT				
Total Sway Area (m^2^/s^4^)	0.476 *	−0.427 *	−0.238	−0.402
Jerk (m^2^/s^5^)	0.486 *	−0.269	−0.345	−0.271
RMS Sway (m/s^2^)	0.331	−0.673 ***	−0.079	−0.201
Condition: Feet apart, eyes closed, firm surface DT				
Total Sway Area (m^2^/s^4^)	0.446 *	−0.478 **	−0.186	−0.304
Jerk (m^2^/s^5^)	0.623 **	−0.427 *	−0.377	−0.321
RMS Sway (m/s^2^)	0.478 *	−0.531 **	−0.170	−0.303
Condition: Feet together, eyes open, firm surface, DT				
Total Sway Area (m^2^/s^4^)	0.520 *	−0.448 *	−0.311	−0.186
Jerk (m^2^/s^5^)	0.595 **	−0.402 *	−0.358	−0.276
RMS Sway (m/s^2^)	0.488 *	−0.438 *	−0.320	−0.205
Condition: Feet together, eyes closed, firm surface, DT				
Total Sway Area (m^2^/s^4^)	0.572 *	−0.551 **	−0.544 *	−0.064
Jerk (m^2^/s^5^)	0.570 *	−0.361	−0.406	−0.071
RMS Sway (m/s^2^)	0.563 *	−0.548 **	−0.541 *	−0.045
Condition: Feet apart, eyes open, firm surface, DTC				
Total Sway Area (m^2^/s^4^)	−0.089	−0.058	−0.005	−0.237
Jerk (m^2^/s^5^)	0.050	0.122	0.074	0.029
RMS Sway (m/s^2^)	−0.069	−0.201	0.001	−0.284
Condition: Feet apart, eyes closed, firm surface, DTC				
Total Sway Area (m^2^/s^4^)	−0.084	0.052	0.310	0.190
Jerk (m^2^/s^5^)	0.168	0.068	0.063	0.046
RMS Sway (m/s^2^)	0.059	−0.001	0.204	−0.010
Condition: Feet together, eyes open, firm surface, DTC				
Total Sway Area (m^2^/s^4^)	0.097	0.205	0.173	0.321
Jerk (m^2^/s^5^)	0.058	0.174	0.140	−0.365
RMS Sway (m/s^2^)	0.099	0.224	0.129	0.241
Condition: Feet together, eyes closed, firm surface, DTC				
Total Sway Area (m^2^/s^4^)	0.043	0.415 *	0.255	0.265
Jerk (m^2^/s^5^)	−0.082	0.434 *	0.391	0.331
RMS Sway (m/s^2^)	0.086	0.328	0.163	0.255
Condition: Feet apart, eyes open, foam surface				
Total Sway Area (m^2^/s^4^)	0.605 **	−0.364 *	−0.392	−0.322
Jerk (m^2^/s^5^)	0.584 **	−0.427 *	−0.491 *	−0.424
RMS Sway (m/s^2^)	0.568 **	−0.386 *	−0.367	−0.320
Condition: Feet apart, eyes closed, foam surface				
Total Sway Area (m^2^/s^4^)	0.518 *	−0.570 **	−0.414	−0.108
Jerk (m^2^/s^5^)	0.350	−0.420 *	−0.422	−0.149
RMS Sway (m/s^2^)	0.522 *	−0.587 **	−0.371	−0.174
Condition: Feet together, eyes open, foam surface				
Total Sway Area (m^2^/s^4^)	0.486 *	−0.631 **	−0.606 *	−0.331
Jerk (m^2^/s^5^)	0.379	−0.446 *	−0.530 *	−0.235
RMS Sway (m/s^2^)	0.463 *	−0.572 **	−0.630 *	−0.301
Condition: Feet together, eyes closed, foam surface				
Total Sway Area (m^2^/s^4^)	−0.265	−0.179	0.267	0.632
Jerk (m^2^/s^5^)	−0.088	−0.357	−0.045	0.158
RMS Sway (m/s^2^)	−0.265	−0.179	0.267	0.632
Condition: Tandem, eyes open				
Total Sway Area (m^2^/s^4^)	0.198	−0.836 **	−0.751	0.387
Jerk (m^2^/s^5^)	0.500	−0.645 *	−0.462	0.323
RMS Sway (m/s^2^)	0.198	−0.800 **	−0.751	0.387

Spearman’s *rho* values with significant *p*-values following FDR corrections (*p* < 0.05) are shown in bold. * *p* < 0.05, ** *p* < 0.01, *** *p* < 0.001. FXTAS-RS, FXTAS Rating Scale; ABC, Activities-specific Balance Confidence Scale; BBS, Berg Balance Scale; FIM, Functional Independence Mobility Scale; MoCA, Montreal Cognitive Assessment; COWAT SS, Controlled Oral Word Association Test standard score, scaled to age and years of education.

## Data Availability

The data presented in this study are available on request from the corresponding author.
